# Effect of Yanghe Pingchuan granules combined with inhaled western medication on clinical efficacy and serum inflammatory cytokine levels in patients with kidney deficiency and phlegm-turbid-type COPD in the stable phase

**DOI:** 10.1097/MD.0000000000045512

**Published:** 2025-10-31

**Authors:** Ranqin Zuo, Yaoyang Zhang, Jiang Wang, Lingqi Kong, Ju Huang, Huizhi Zhu

**Affiliations:** a Department of Critical Care Medicine, Changzhou Jintan District Hospital of Traditional Chinese Medicine, Changzhou, Jiangsu Province, China; b Department of Respiratory Medicine, The First Affiliated Hospital of Anhui University of Traditional Chinese Medicine, Hefei, Anhui Province, China.

**Keywords:** COPD in the stage phase, kidney deficiency and phlegm turbidity type, Yanghe Pingchuan granules

## Abstract

To explore the effects of Yanghe Pingchuan granules combined with inhaled Western medication on the clinical efficacy and serum inflammatory cytokine levels in patients with kidney deficiency and phlegm-turbid-type chronic obstructive pulmonary disease (COPD) in the stable phase. Using the retrospective analysis method, we collected 108 patients with kidney deficiency and phlegm-turbid-type COPD in the stable phase admitted to the First Affiliated Hospital of Anhui University of Traditional Chinese Medicine from January 2020 to December 2021, and divided them into a combined group and a control group according to medication method, with 56 cases in the combined group and 52 in the control group. In the control group, Budesonide and Formoterol Dry Powder Inhaler was given, and in the combined group, Yanghe Pingchuan granules were added on that basis, with a treatment course of 2 weeks. We compared the TCM syndrome score, post-treatment efficacy, pulmonary functions (forced expiratory volume in one second [FEV1], forced vital capacity [FVC], FEV1/FVC, distance of six-minute walking test [6MWT]), COPD assessment test (CAT) score, and changes in serum inflammatory cytokines (IL-6, IL-10, IL-17, LTB4) levels in the 2 groups. After treatment, the total effective rate of the combined group was 91%, significantly higher than that of the control group, which was 75% (*P* < .05). After the treatment, the TCM syndrome score, pulmonary functions (FEV1, FVC, FEV1/FVC, 6MWT), CAT score, and changes in serum inflammatory cytokines (IL-6, IL-10, IL-17, LTB4) levels in the 2 groups decreased compared with those before treatment (*P* < .01), and all of the above indexes, except the pulmonary function FEV1/FVC ratio, in the combined group were better than those in the control group (*P* < .05). Yanghe Pingchuan granules combined with inhaled Western medication has better clinical efficacy than single inhaled Western medication in patients with kidney deficiency and phlegm-turbid-type COPD in the stable phase, by significantly improving clinical symptoms, enhancing the pulmonary functions, and reducing the inflammation level.

## 1. Introduction

Chronic obstructive pulmonary disease (COPD) is a chronic disease characterized by persistent airflow limitation and respiratory symptoms, which are clinically manifested as cough, sputum, wheezing, shortness of breath, palpitations, and chest tightness. As a common disease endangering human life and health, the prevention and treatment of COPD during its stable phase is critical.^[1]^ Currently, one of the main Western medicine drugs used to treat COPD in the stable phase is Budesonide and Formoterol Dry Powder Inhaler, the main components of which are budesonide (an inhaled corticosteroid) and formoterol (a long-acting β2-agonist). Studies showed that this drug can significantly improve clinical symptoms and enhance pulmonary function indexes in patients with stable-phase COPD.^[2,3]^ According to the traditional Chinese medicine (TCM) theory, COPD is caused by a disease of the lungs, being chronic and refractory. According to TCM, symptoms such as wheezing and shortness of breath are closely related to lung and kidney deficiencies and the kidneys’ failure to hold *qi*, while ineffective cough may lead to phlegm and blood stasis blocking the lung channels. Therefore, the kidney deficiency and phlegm turbidity type is a common syndrome pattern in the stable stage of COPD.^[4,5]^ In response to this pattern, the First Affiliated Hospital of Anhui University of Chinese Medicine developed Yangpingping Chuan Granules, a traditional Chinese medicine formulation composed of Asarum, White Mustard Seed, Corydalis, Astragalus, Platycodon, Prepared Rehmannia, Epimedium, Schisandra, and Angelica. These granules are believed to tonify the kidney, promote blood circulation, regulate qi and blood, and resolve phlegm to alleviate coughing. They have demonstrated notable therapeutic effects in treating lung diseases associated with kidney deficiency and phlegm-dampness. Previous studies^[6]^ employed thin-layer chromatography to identify the main components of Yangpingping Chuan Granules, including ephedrine hydrochloride, schisandrin, and sinigrin thiocyanate. Pharmacological experiments further demonstrated that these granules could alleviate rapid breathing and coughing in asthma model rats, while also reducing inflammation markers. However, its application experience in the stable phase of COPD is limited.^[7,8]^ Based on the above background, this study adopted a retrospective analysis method, aiming to investigate the effects of Yanghe Pingchuan granules combined with inhaled Western medication on the clinical efficacy and serum inflammatory cytokine levels in patients with kidney deficiency and phlegm-turbid-type COPD in the stable phase, in order to provide more reference for clinical treatment. The study details are reported as follows.

## 2. Methods

### 2.1. General data

Using the retrospective analysis method, we collected 108 patients with kidney deficiency and phlegm-turbid-type COPD in the stable phase admitted to the First Affiliated Hospital of Anhui University of Traditional Chinese Medicine from January 2020 to December 2021, and divided them into a combined group and a control group according to medication method, with 56 cases in the combined group and 52 in the control group. Specifically, there were 13 female patients and 39 male ones in the control group, aged (72.42 ± 7.71), and 11 female ones and 45 male ones in the combined group, aged (71.29 ± 6.23). There was no statistically significant difference in the comparison of the basic data of the patients in the 2 groups (*P* > .05), and they were comparable, as shown in Table 1. This study was examined and approved by the Ethics Committee of the First Affiliated Hospital of Anhui University of Traditional Chinese Medicine (Approval No.: 2018-AH06).

**Table 1:** Basic data comparison of the 2 groups of patients with kidney deficiency and phlegm-turbid-type COPD in the stable phase.

	Combined group (n = 56)	Control group (n = 52)	*P*	Effect size (95% CI)
Age (yr)	71.29 ± 6.23	72.42 ± 7.71	.403	Cohen *d* = −0.16 (95% CI: −0.54–0.22)
Gender (F/M)	11/ 45	13/ 39	0.662	OR = 0.74 (95% CI: 0.29–1.88)

CI = confidence interval, COPD = chronic obstructive pulmonary disease.

### 2.2. Case selection

#### 2.2.1. Diagnosis and inclusion criteria

Western medicine diagnostic criteria for COPD refer to the “Guidelines for the Diagnosis and Treatment of COPD (2021 Revision),”^[1]^ and for diagnosis criteria for stable-phase COPD refer to the discharge criteria in “Guidelines for the Diagnosis and Treatment of Community-acquired Pneumonia.”^[7]^ The criteria are as follows. Body temperature ≤ 37.0°C; Heart rate ≤ 100 beats/min at rest; Respiratory rate ≤ 24 beats/min at rest; Systolic blood pressure ≥ 90 mm Hg; Arterial oxygen saturation ≥ 90% or finger pulse oximetry ≥ 60 mm Hg without oxygen; Inhalation of SABA should be less than once every 4 hours; Clinically stable for 12 to 24 hours; Leukocyte count < 10 × 10^9^/L or neutrophil percentage ≤ 70%; No need to use antimicrobial drugs; and The patient is able to eat, and sleep is not affected by the respiratory distress.

The diagnostic criteria for traditional Chinese medicine syndromes are based on the relevant sections of the “Guidelines for the Diagnosis and Treatment of COPD in Traditional Chinese Medicine (2011 Edition)”^[9,10]^ and the “Guidelines for the Diagnosis and Treatment of COPD in Integrated Chinese and Western Medicine (2022 Edition).”^[11]^ For the syndrome of kidney deficiency and phlegm-dampness, the criteria include: Cough, expectoration, chest tightness; Wheezing, shortness of breath, fatigue or spontaneous sweating, aggravated by physical activity; Susceptibility to colds, aversion to wind; Low back and knee soreness; Tinnitus, dizziness, or facial puffiness; Frequent urination, increased nighttime urination, or incontinence during coughing; and Pale tongue with a white or greasy coating, or a deep, thin, or weak pulse. Diagnosis is confirmed when at least 2 symptoms from 1st, 2nd, or 3rd are present, along with at least 2 symptoms from 4th, 5th, 6th, or 7th.

Inclusion criteria: The patient meets the diagnostic criteria of stable-phase COPD of a kidney deficiency and phlegm turbidity type, and has no acute exacerbation in the last 3 months.

#### 2.2.2. Exclusion criteria

Patients not meeting the inclusion criteria of 1.2.1; Patients with severe respiratory comorbidities (such as bronchiectasis, pleural effusion, and tumors) that could confound the study observations; Patients with mental illness; Pregnant and breastfeeding patients; Patients with severe dysfunction of the heart, brain, liver, or kidneys, or other systemic diseases that could confound the study observations; Glucocorticosteroid-using patients; and Patients allergic to Yanghe Pingchuan granules.

### 2.3. Treatment

Both groups received conventional treatments including oxygen therapy, anti-infective agents, antitussives, expectorants, and other standard care. The control group received budesonide/formoterol dry powder inhaler (Ⅱ) (produced by AstraZeneca AB, Cambridge, United Kingdom) in addition to conventional treatment, one inhalation each time, twice a day. The combination group was given Yanghe Pingchuan granules (10 g/bag, containing 2.7 g of raw herb per gram, provided by the Preparation Center of the First Affiliated Hospital of Anhui University of Traditional Chinese Medicine, production lot number: 20190718) on the basis of the treatment for the control group. The specific formula is: 15 g of prepared rehmannia root, 10 g of morinda root, 6 g of schisandra berry, 10 g of Chinese angelica root, 6 g of ephedra, 10 g of white mustard seed, 10 g of lepidium seed, 9 g of inula flower, and 10 g of platycodon root. Take the medication twice daily, half an hour after breakfast and dinner, with warm water, for a 2-week course.^[2,11]^ Various outcome measures were detected and recorded in detail at the beginning and the end of the clinical study.

### 2.4. Outcome measures

#### 2.4.1. TCM syndrome score

The TCM syndrome score table was developed referring to the “Guiding Principles for Clinical Research of New Chinese Medicines.”^[12]^ The primary symptoms include cough, wheezing, shortness of breath, sputum expectoration, and chest tightness, with 0, 2, 4, 6 indicating the severity score. The secondary symptoms include palpitations, aversion to cold with cold limbs, dizziness, tinnitus, generalized edema, frequent urination, fatigue with spontaneous sweating, and poor appetite, with 0, 1, 2, 3 indicating the severity score. Lower final scores indicated more significant improvement in TCM symptoms.

#### 2.4.2. Clinical efficacy

Referring to the “Guidelines for Clinical Research of New Chinese Medicines,”^[12]^ a reduction of more than 70% in TCM syndrome score was considered as marked effectiveness, a reduction of more than 30% was considered as effectiveness, and a reduction of <30% was considered as ineffectiveness. The total effective rate was the ratio of the sum of marked effectiveness and effectiveness to the total number of cases in each group.

#### 2.4.3. Pulmonary function indexes

Use an EasyOne Spirometer (Bry-Air, Inc., Sunbury) to detect the forced expiratory volume in one second (FEV1) and forced vital capacity (FVC) before treatment and after 2 weeks of treatment in both groups, and the FEV1/FVC ratio was calculated. The distance of six-minute walking test (6MWT) before treatment and after 2 weeks of treatment in both groups was recorded.

#### 2.4.4. COPD assessment test (CAT) score^[13]^

The CAT score assesses the improvement in the quality of survival of the patients with a total score of 40. The lower the score, the more significant the improvement in the quality of survival of the patients.

#### 2.4.5. Serum inflammatory cytokines

Venous blood samples (5 mL) were collected from patients before treatment and after 4 weeks of treatment. The samples were centrifuged at 3000*g* for 5 minutes to separate serum. Serum levels of IL-6, IL-10, IL-17, and LTB4 were measured using enzyme-linked immunosorbent assay kits (Keshuo Biotechnology Co., Ltd., Jinan, Shandong Province, China) following the manufacturer’s instructions.

### 2.5. Statistical methods

This study initially performed a normality test on all continuous variables using the Shapiro–Wilk test to determine if the data followed a normal distribution. For normally distributed variables, the mean ± standard deviation was used for description. Independent samples *t*-tests were employed for between-group comparisons, while paired *t*-tests were used for within-group pre- and post-treatment comparisons. For non-normally distributed variables, non-parametric tests, such as the Mann–Whitney *U* test, were applied. The effect size was calculated using Cohen *d* to assess the magnitude of between-group differences. Clinical efficacy was compared using odds ratios and relative risks, with 95% confidence intervals reported. All hypothesis tests were two-tailed, with a significance level of *P* < .05. The Bonferroni correction method was applied to adjust *P*-values during multiple comparisons of cytokine levels, reducing Type I errors. All statistical analyses were performed using SPSS software.

## 3. Results

### 3.1. Comparison of TCM syndrome scores

At baseline, there were no significant differences in TCM syndrome scores between the 2 groups (*P* > .05), confirming comparability.

After treatment, both groups showed statistically significant reductions in TCM syndrome scores compared to baseline (intra-group *P* < .01), with that of the combined group lower than that of the control group (inter-group *P* < .01) (Table 2).

**Table 2:** Comparison of TCM syndrome score between the combined group and the control group.

Group	n	Before (mean ± SD)	After (mean ± SD)	MD (95% CI)	*P*	Effect size (95% CI)
Combined group	56	29.07 ± 6.53	16.30 ± 2.06	−12.77 (−14.23–−11.31)	<.001	–
Control group	52	29.12 ± 7.01	20.08 ± 3.49	−9.04 (−10.18–−7.90)	<.001	–
Between groups	–	–	–	−3.73 (−5.61–−1.85)	<.001	Cohen d = −0.75 (95% CI −1.14–−0.36)

CI = confidence interval, SD = standard deviation, TCM = traditional Chinese medicine.

### 3.2. Comparison of clinical efficacy

After treatment, the total effective rate in the combination group (91%) was significantly higher than that in the control group (75%) (*P* < .05) (Table 3).

**Table 3:** Comparison of clinical efficacy between the combined group and the control group.

Group	n	Marked effectiveness	Effectiveness	Ineffectiveness	Total effective rate	OR (95% CI)	RR (95% CI)	*P*
Combined group	56	13 (23.2%)	38 (67.9%)	5 (8.9%)	91.1%	3.31 (1.13–11.3)	2.8 (1.07–7.31)	.037
Control group	52	7 (13.5%)	32 (61.5%)	13 (25.0%)	75.0%			

CI = confidence interval, OR = odds ratios, RR = relative risks.

### 3.3. Comparison of pulmonary functions

At baseline, there were no statistically significant differences in FEV1, FVC, FEV1/FVC ratio, and 6MWT between the 2 groups (*P *> .05).

After treatment, both groups showed significant improvements in FEV1, FVC, FEV1/FVC ratio, and 6MWT compared to baseline (intra-group *P* < .01). However, the combined group demonstrated superior improvements in FEV1, FVC, and 6MWT compared to the control group (*P* < .01), while no significant inter-group difference was observed in the FEV1/FVC ratio (*P* > .05) (Table 4).

**Table 4:** Comparison of pulmonary functions between the combined group and the control group.

Group	Before (mean ± SD, 95% CI)	After (Mean ± SD, 95% CI)	Difference (95% CI)	*t*	*P*	Effect size (Cohen *d*)
FEV1 (L)						
Combined group	0.98 ± 0.21	1.49 ± 0.26	0.51 (0.29–0.73)	−2.737	.006	0.10 (0.06–0.13)
Control group	0.96 ± 0.18	1.33 ± 0.34	0.37 (0.17–0.57)			
FVC (L)						
Combined group	1.75 ± 0.28	2.17 ± 0.14	0.42 (0.23–0.61)	3.44	.001	−0.33 (−0.56–−0.11)
Control group	1.83 ± 0.20	2.02 ± 0.19	0.19 (0.06–0.32)			
FEV1/FVC (%)						
Combined group	52.23 ± 11.08	58.96 ± 6.01	6.73 (2.84–10.62)	0.216	.829	0.35 (0.14–0.56)
Control group	48.85 ± 7.53	56.92 ± 10.15	8.07 (5.23–10.91)			
6MWT (m)						
Combined group	293.77 ± 18.14	338.13 ± 16.16	44.36 (35.56–53.16)	6.60	.001	0.18 (0.07–0.29)
Control group	290.55 ± 18.16	308.00 ± 18.82	17.45 (6.63–28.27)			

6MWT = distance of six-minute walking test, CI = confidence interval, FEV1 = forced expiratory volume in one second, FVC = forced vital capacity, SD = standard deviation.

### 3.4. Comparison of CAT score

At baseline, there were no significant differences in CAT scores between the 2 groups (*P* > .05), confirming comparability.

After treatment, both groups showed statistically significant reductions in CAT scores compared to baseline (intra-group *P* < .01), with that of the combined group lower than that of the control group (inter-group *P* < .01) (Table 5).

**Table 5:** Comparison of CAT scores between the combined group and the control group.

Group	n	Before (mean ± SD)	After (mean ± SD)	*t*	*P*	Effect size (95% CI)
Combined group	56	20.56 ± 2.10	10.87 ± 1.59	−8.983	.000	Cohen *d* = −0.29 (−0.67–0.09)
Control group	52	21.25 ± 2.71	13.67 ± 1.65			

CAT = COPD assessment test, CI = confidence interval, SD = standard deviation.

### 3.5. Comparison of serum inflammatory cytokine levels

At baseline, there were no statistically significant differences in serum levels of IL-6, IL-10, IL-17, and LTB4 between the 2 groups (*P* > .05).

After treatment, both groups exhibited significant reductions in serum IL-6, IL-17, and LTB4 levels compared to baseline (intra-group *P* < .01), along with increased serum IL-10 levels (intra-group *P* < .01). Notably, the combined group demonstrated more pronounced reductions in IL-6, IL-17, and LTB4 (inter-group *P* < .01) and a greater elevation in IL-10 (inter-group *P* < .01) compared to the control group (Table 6).

**Table 6:** Comparison of serum inflammatory cytokine levels between the combined and the control group.

	Combined group (n = 56)		Control group (n = 52)		*t*	*P*	95% CI	Effect size (Cohen *d*)	Adjusted *P*-value (multiple comparisons)
	Before (mean ± SD)	After (mean ± SD)	Before (mean ± SD)	After (mean ± SD)					
IL-6 (ng/L)	68.92 ± 14.10	19.88 ± 3.05	71.99 ± 11.53	27.40 ± 5.15	5.094	.001	−52.90 (−58.62–−47.18)	1.12 (0.85–1.39)	.001
IL-10 (pg/mL)	48.40 ± 10.28	159.14 ± 20.06	50.51 ± 18.39	141.14 ± 11.24	−3.202	.001	−110.48 (−116.56–−104.40)	0.91 (0.68–1.14)	.002
IL-17 (pg/mL)	406.31 ± 117.39	226.59 ± 12.25	399.95 ± 124.87	262.99 ± 10.16	6.064	.000	−179.76 (−195.89–−163.63)	0.85 (0.67–1.03)	.001
LTB4 (ng/mL)	77.90 ± 24.55	42.68 ± 1.44	75.71 ± 23.16	46.81 ± 1.16	−11.163	.000	−35.22 (−39.19–−31.25)	1.16 (0.89–1.43)	.001

CI = confidence interval, SD = standard deviation.

## 4. Discussion

COPD is a prevalent respiratory disorder characterized by recurrent acute exacerbations and high mortality rates, imposing a substantial burden on both individual patients and society.^[14]^ While Western medicine remains a cornerstone in COPD management, its limited efficacy and frequent side effects associated with long-term use may compromise medication adherence, thereby diminishing therapeutic outcomes.^[11]^ In recent years, TCM has gained increasing recognition in COPD clinical practice and has demonstrated promising efficacy. Studies have shown that integrating Chinese herbal medicine with Western therapeutics can significantly enhance treatment effectiveness and improve prognosis in COPD patients.

According to TCM theory, COPD falls under the categories of “Fei Zhang” (lung distention) and “Chuan Zheng” (panting syndrome), characterized by a chronic and refractory nature primarily attributed to deficiency of the lung, spleen, and kidneys. Dysfunction of the lung’s diffusion and descent functions, the spleen’s transformation and transportation, and the kidney’s qi transformation may lead to stagnation of turbid phlegm and blood stasis in the lung collaterals, causing systemic visceral dysfunction. Among COPD-related TCM syndromes, phlegm-heat obstructing the lung and lung-kidney qi deficiency are the predominant syndrome patterns.^[4,5]^ The kidney-deficiency with phlegm-turbidity pattern is characterized by cough, sputum production, and chest tightness (oppression). Wheezing and dyspnea may occur, accompanied by fatigue or spontaneous sweating. Symptoms typically worsen with exertion. Patients are often prone to catching colds and report an aversion to wind. Soreness and weakness of the lumbar region and knees are common. Tinnitus, dizziness, and facial puffiness may be present. Urinary frequency, increased nocturia, and stress incontinence triggered by coughing can also occur. The tongue is usually pale with a white or greasy-white coating. The pulse is typically deep and thin (thready/weak). Yanghe Pingchuan granules, a compound herbal formula, are designed to tonify the kidney, activate blood circulation, regulate nutrient qi, and resolve phlegm. The formula synergistically combines: *Ephedra sinica, Sinapis alba, Lepidium apetalum, Inula japonica*, and *Platycodon grandiflorus* to diffuse the lung, relieve wheezing, eliminate phlegm, and resolve retained fluid; *Rehmannia glutinosa* praeparata, *Morinda officinalis*, and *Schisandra chinensis* to tonify kidney yang, warm the organs, and transform turbid phlegm; and *Angelica sinensis* to activate blood circulation, resolve stasis, and improve lung collateral stasis.^[15]^

In this study, patients with kidney deficiency and phlegm-turbid-type COPD in the stable phase were treated with Yanghe Pingchuan granules combined with budesonide formoterol. The results showed that the total effective rate of the combined group was significantly higher than that of the control group, and outperformed the control group in terms of improvement of TCM symptoms and CAT score. This indicates that Yanghe Pingchuan granules can effectively alleviate symptoms such as cough, expectoration, wheezing, shortness of breath, dizziness, tinnitus, and edema in COPD patients with kidney deficiency and phlegm-turbidity syndrome, while significantly improving their quality of life. Pharmacological studies further corroborated these findings: Ephedrine derived from *E sinica* (ephedra) demonstrates anti-asthmatic and antitussive effects.^[14]^ *Descurainiae Semen* (Lepidii/Descurainiae seeds) contains bioactive constituents including cardiac glycosides, flavonoids, and organic acids, which exhibit bronchodilator, diuretic, expectorant, and antitussive properties.^[6,16,17]^ *S alba* (white mustard seeds), *P grandiflorus* (balloon flower root), and *S chinensis* (magnolia berry) demonstrate synergistic expectorant and cough-suppressing effects.^[18–21]^

The study also demonstrated significant improvements in FEV1, FVC, and 6MWT (6-Minute Walk Test) across both treatment groups, with the combined group showing superior pulmonary function enhancement compared to the control group. However, no statistically significant inter-group differences were observed in FEV1/FVC ratio improvement. This suggests that Yanghe Pingchuan granules combined with inhaled Western medication may outperform monotherapy in optimizing pulmonary function parameters. COPD is pathologically characterized by progressive airway obstruction and pulmonary function impairment, primarily attributable to chronic tobacco smoking or exposure to noxious gases and particulate matter. These factors induce persistent airway inflammatory responses and structural remodeling, manifested as thickened airway walls and disrupted elastic fibers, which further exacerbate airflow limitation and accelerate pulmonary function decline.^[22]^ Yanghe Pingchuan granules exhibit kidney-tonifying, blood-activating, and phlegm-resolving properties, effectively addressing pulmonary and renal deficiency syndromes. This therapy alleviates symptoms such as dyspnea, wheezing, and cough, while simultaneously fortifying the spleen-stomach system, nourishing the 5 viscera, and enhancing systemic resilience, thereby improving clinical outcomes and pulmonary functions.

The post-treatment analysis revealed that the combined group demonstrated significant reductions in serum pro-inflammatory cytokines (IL-6, IL-17, and LTB) alongside a marked elevation in anti-inflammatory IL-10 levels compared to the control group. These results indicate that Yanghe Pingchuan granules combined with inhaled Western medication exerts superior anti-inflammatory effects. COPD is characterized by chronic inflammation in the airways and lung tissues, leading to thickening of airway walls and hyperplasia of mucous glands, further leading to airway obstruction, luminal narrowing, and hypercontractility of airway smooth muscle to restrict airflow and exacerbate pulmonary injury.^[23]^ IL-6, IL-17, and LTB4 function as key pro-inflammatory cytokines that critically regulate inflammatory and immune processes. These mediators can activate inflammatory cascades by promoting the release of secondary inflammatory mediators, sustain chronic inflammation through proliferation and activation of inflammatory cells, amplifying airway inflammation. They may also induce and orchestrate the production of downstream inflammatory mediators, exacerbating inflammatory responses. Pharmacological studies revealed that active components of *R glutinosa* praeparata, *M officinalis*, and *A sinensis* exert anti-inflammatory effects, potentially explaining the superior anti-inflammatory efficacy of combination therapy compared to Western monotherapy.^[24–26]^

In conclusion, the combination of Yangpingping Chuan Granules and Western inhalation therapy for patients with stable-phase kidney deficiency and phlegm-dampness type COPD is more effective than Western medicine alone in improving clinical symptoms, enhancing lung function, and reducing inflammation levels. However, this study was retrospective and single-center, with a relatively small sample size (n = 108). A two-week treatment period was chosen based on the onset window reported in prior studies^[2,11]^ and to maximize case retrieval from the electronic medical record system. These design features may introduce inherent bias: most cases were drawn from a regional Class A tertiary hospital, likely underrepresenting patients treated at primary-level hospitals. Accordingly, the findings are preliminary and require confirmation in prospective studies. Future research should include multicenter, adequately powered prospective trials and mechanistic investigations to clarify the actions of Yanghe Pingchuan Granules, thereby strengthening the evidence base for integrative Chinese–Western therapy in COPD.

## Author contributions

**Conceptualization:** Yaoyang Zhang.

**Data curation:** Ranqin Zuo, Yaoyang Zhang, Ju Huang.

**Formal analysis:** Ranqin Zuo, Jiang Wang.

**Funding acquisition:** Jiang Wang, Ju Huang.

**Investigation:** Jiang Wang, Lingqi Kong, Huizhi Zhu.

**Methodology:** Ranqin Zuo, Huizhi Zhu.

**Project administration:** Yaoyang Zhang, Jiang Wang, Huizhi Zhu.

**Resources:** Lingqi Kong.

**Software:** Lingqi Kong.

**Supervision:** Lingqi Kong, Ju Huang.

**Validation:** Huizhi Zhu.
